# Association between county-level risk groups and COVID-19 outcomes in the United States: a socioecological study

**DOI:** 10.1186/s12889-021-12469-y

**Published:** 2022-01-13

**Authors:** Sadiya S. Khan, Amy E. Krefman, Megan E. McCabe, Lucia C. Petito, Xiaoyun Yang, Kiarri N. Kershaw, Lindsay R. Pool, Norrina B. Allen

**Affiliations:** 1 Division of Cardiology, Department of Medicine, Northwestern University Feinberg School of Medicine, 680 N. Lake Shore Drive Ste. 1400, Chicago, IL 60611 USA; 2 Department of Preventive Medicine, Northwestern University Feinberg School of Medicine, Chicago, IL USA

**Keywords:** COVID-19, County-level health, Communicable diseases, Pandemic

## Abstract

**Background:**

Geographic heterogeneity in COVID-19 outcomes in the United States is well-documented and has been linked with factors at the county level, including sociodemographic and health factors. Whether an integrated measure of place-based risk can classify counties at high risk for COVID-19 outcomes is not known.

**Methods:**

We conducted an ecological nationwide analysis of 2,701 US counties from 1/21/20 to 2/17/21. County-level characteristics across multiple domains, including demographic, socioeconomic, healthcare access, physical environment, and health factor prevalence were harmonized and linked from a variety of sources. We performed latent class analysis to identify distinct groups of counties based on multiple sociodemographic, health, and environmental domains and examined the association with COVID-19 cases and deaths per 100,000 population.

**Results:**

Analysis of 25.9 million COVID-19 cases and 481,238 COVID-19 deaths revealed large between-county differences with widespread geographic dispersion, with the gap in cumulative cases and death rates between counties in the 90^th^ and 10^th^ percentile of 6,581 and 291 per 100,000, respectively. Counties from rural areas tended to cluster together compared with urban areas and were further stratified by social determinants of health factors that reflected high and low social vulnerability. Highest rates of cumulative COVID-19 cases (9,557 [2,520]) and deaths (210 [97]) per 100,000 occurred in the cluster comprised of rural disadvantaged counties.

**Conclusions:**

County-level COVID-19 cases and deaths had substantial disparities with heterogeneous geographic spread across the US. The approach to county-level risk characterization used in this study has the potential to provide novel insights into communicable disease patterns and disparities at the local level.

**Supplementary Information:**

The online version contains supplementary material available at 10.1186/s12889-021-12469-y.

## Introduction

Since the first case of the coronavirus disease 2019 (COVID-19) was confirmed in the United States (US) in Snohomish county, Washington, on January 21^st^, 2020 [1, 2], the US has experienced more cases (99,467 cases per million population) and deaths (585,708) than any other country in the world (as of May 15, 2021) [3]. Clinical and epidemiologic data have identified several modifiable health factors (e.g., obesity [4], hypertension [5], cardiovascular disease [6]) as key risk factors of adverse outcomes such as hospitalization and mortality related to severe COVID-19 infection [7, 8]. However, assessment of individual-level health factors incompletely characterizes risk related to underlying social determinants of health that may contribute to observed differences in COVID-19 outcomes [9, 10].

Understanding how place-based disadvantage has influenced COVID-19 case and death rates is critical for developing strategies to address long-term sequalae of COVID-19 [11] and to target vaccination efforts [12] at the local level. This is especially important given the significant geographic heterogeneity observed in the burden of COVID-19 with large differences in morbidity and mortality among counties within the US [13, 14]. Several studies have focused on single social and environmental factors (e.g., housing overcrowding [15], air pollution [16]) or grouped factors based on existing county-level rankings (e.g., social vulnerability index [17]) to examine differences at the county-level in COVID-19 outcomes. However, susceptibility to COVID-19 infection and mortality is likely due to interrelated biological, social, and environmental factors; to date, no studies have developed a comprehensive measure that integrates place-based factors across multiple domains to classify county-level risk for COVID-19 outcomes.

This study sought to use a novel method for clustering, latent class analysis, to identify meaningful groups of counties based on county-level characteristics across multiple sociodemographic, health, and environmental domains and to examine associations with COVID-19 cases and deaths.

## Methods

### Data Sources

We harmonized and linked a comprehensive set of county-level measures from a variety of resources including the U.S. Census Bureau, County Health Rankings (CHR), Centers for Disease Control and Prevention (CDC) Wide-ranging Online Data for Epidemiologic Research (WONDER) mortality database, Area Health Resources Files (AHRF), and the United States Department of Agriculture (USDA) across all available counties coded by Federal Information Processing Standard county codes for a total of 30 variables (Supplemental Table 1). First, from these various data sources, we obtained relevant measures to characterize counties’ profiles according to the following categories based on pre-pandemic data on demographics, socioeconomic status, health status, healthcare access, and environmental factors. Next, we queried county-level COVID-19 data on 2/18/21 to include all deaths and cases from 1/21/20 to 2/17/21 from The New York Times GitHub where information on cases and fatalities is updated daily (https://github.com/nytimes/COVID-19-data) to link with our integrative county-level index. For a subset of counties, vaccination data (number of first dose and two doses administered) were manually abstracted from county and state department of public health websites by SSK, AEK, LRP, and NBA. All data were deidentified and publicly available, and therefore deemed exempt from Northwestern University’s Institutional Review Board.

### Measures

Counties were described using metrics relating to residents’ demographics, socioeconomic status, health status, healthcare access, as well as environmental factors (Supplemental Table 1). Demographic factors included proportion of racial/ethnic minority and older residents in addition to population density and urbanization status. Socioeconomic metrics included rates of varying educational levels, poverty, and housing. Health status included quality of life and morbidity and mortality rates. Healthcare access reflected insurance, hospital beds per capita, and primary care physician density. Finally, the environmental factor was a measure of air pollution. The primary outcomes were cumulative deaths and cases per 100,000 population attributed to COVID-19 at the county-level. As an exploratory outcome, COVID-19 vaccine doses administered in a subset of counties was examined.

### Statistical Analysis

We included all counties for which we had complete ecological data across county-level metrics that span demographic, socioeconomic status, health status, healthcare access, and environmental factors and at least 5 COVID-19 deaths. We used latent class analysis (LCA) [18] to classify US counties with others who have similar socioecological profiles based on these included metrics. These groupings represent meaningful clusters defined by a combination of all the observed variables by using model-based predicted posterior probabilities. The LCA model incorporated binary and ordinal variables as-is, and continuous variables were categorized as tertiles before inclusion. The model was fit using R package poLCA, which estimates the latent class model by maximizing a log-likelihood function using the EM algorithm. We set the max iterations to 10000. To select the optimal number of classes (between 1-5) for the final LCA model, we imposed the following criteria based on previously published methods: (1) qualitative evaluation of distinct predicted classes or groups, (2) ensuring no group had fewer than 5% of counties, and (3) minimizing the Bayesian information criterion. Model fit statistics for models with 1-5 latent classes (Log-likelihood, Residual DF, BIC, aBIC, cAIC, and Likelihood-Ratio) in Supplemental Table 2 [19]. Conditional probabilities and a classification table for each of the county-level variables for the final model are shown in Supplemental Tables 3 and 4.

For the final identified groups, we described county-level characteristics using means, medians, and proportions, as appropriate, and described crude rates of cumulative COVID-19 cases and deaths per 100,000 population. We additionally identified the ten counties in each group with the highest deaths per 100,000 population to describe dynamic trends in cumulative deaths since the beginning of the pandemic and cumulative cases, deaths, and vaccination rates per 100,000.

In the primary analysis, we calculated the association between each county-level risk group and each outcome (COVID-19 deaths and cases) using linear regression models. We fit two sets of models: 1) unadjusted and 2) adjusted for the time since the first confirmed case in the county, to allow comparison between counties at similar points in their outbreak. Point estimates were interpreted in context of their magnitude and Wald-style 95% confidence interval. We completed a sensitivity analysis including all counties reporting at least one COVID-19 death in order to examine the robustness of our estimates (*N*=2694 counties). All analyses were completed using R version 3.6.3 or newer.

## Results

### County-Level Risk Groups

Data on the 30 county-level measures were available for 2,701 US counties. We identified four distinct groups of counties based on demographic, socioeconomic, health status, healthcare access, and environmental profiles (Table 1, Supplemental Figure 1): 1) diverse urban counties with greater social and health assets; 2) rural counties with social and health vulnerabilities; 3) older rural counties with greater social assets; and 4) urban counties with social vulnerabilities. Diverse urban counties had the highest population density (757 [2244]), greatest proportion of at least some college education (71% [7]), lowest rates of unemployment (3% [1]) and percent of children in poverty (11% [4]), and fewest absolute years of potential life lost (5756 [1127] years). By contrast, vulnerable rural counties had the highest proportion of non-Hispanic black residents (17% [19]), lowest proportion of some college education (48% [8]), highest rates of unemployment (5% [1]) and percent of children in poverty (30% [7]), and greatest absolute years of potential life lost (10,768 [2062] years).

**Table 1:** County-level demographic, socioeconomic, health status, healthcare access, and other factors in each county-level risk group in the United States (*N*=2701 counties)

	Diverse Urban counties with greater social assets	Rural Counties with social and medical vulnerabilities	Older Rural counties with greater social assets	Urban Counties with social vulnerabilities
	*n*=461	*n*=947	*n*=712	*n*=581
**Demographics**				
Population density	756.84 (2243.99)	92.35 (447.56)	29.20 (25.03)	378.82 (897.47)
Proportion male	0.50 (0.01)	0.50 (0.03)	0.51 (0.02)	0.50 (0.02)
Proportion non-Hispanic White	0.76 (0.16)	0.69 (0.23)	0.85 (0.14)	0.76 (0.17)
Proportion non-Hispanic Black	0.06 (0.07)	0.17 (0.19)	0.02 (0.03)	0.11 (0.11)
Proportion Hispanic	0.11 (0.10)	0.09 (0.16)	0.09 (0.13)	0.09 (0.12)
Proportion 60+ years old	0.23 (0.05)	0.26 (0.04)	0.29 (0.06)	0.24 (0.04)
Proportion 80+ years old	0.04 (0.01)	0.04 (0.01)	0.05 (0.01)	0.04 (0.01)
Rural-Urban Continuum Code (%)				
1	212 (46.0)	36 ( 3.8)	8 ( 1.1)	155 (26.7)
2	100 (21.7)	86 ( 9.1)	28 ( 3.9)	152 (26.2)
3	74 (16.1)	82 ( 8.7)	52 ( 7.3)	122 (21.0)
4	31 ( 6.7)	72 ( 7.6)	33 ( 4.6)	77 (13.3)
5	14 ( 3.0)	35 ( 3.7)	28 ( 3.9)	12 ( 2.1)
6	10 ( 2.2)	301 (31.8)	210 (29.5)	57 ( 9.8)
7	15 ( 3.3)	158 (16.7)	225 (31.6)	5 ( 0.9)
8	2 ( 0.4)	83 ( 8.8)	56 ( 7.9)	1 ( 0.2)
9	3 ( 0.7)	94 ( 9.9)	72 (10.1)	0 ( 0.0)
**Socioeconomic Status**				
High school graduation rate	88.70 (6.25)	87.97 (7.51)	89.14 (6.99)	88.70 (5.34)
Percent some college education	71.09 (7.23)	48.48 (8.24)	59.89 (9.31)	59.30 (7.61)
Percent unemployed	3.31 (0.74)	4.96 (1.44)	3.80 (1.44)	4.04 (0.91)
Percent children in poverty	11.27 (4.08)	29.85 (7.02)	17.47 (4.90)	19.16 (4.30)
Income ratio (80th to 20th percentile)	4.28 (0.66)	4.99 (0.78)	4.15 (0.43)	4.45 (0.59)
Percent households with overcrowding	2.22 (1.58)	2.75 (1.96)	2.02 (1.54)	2.22 (1.48)
Median income	71557.97 (14989.20)	40188.09 (6170.90)	52465.77 (6985.36)	53390.72 (6798.91)
**Health Status**				
Years of potential life lost rate	5755.71 (1127.19)	10767.91 (2061.60)	7410.08 (1550.12)	8334.47 (1203.43)
Percent reporting fair/poor health	13.56 (2.12)	22.78 (3.62)	15.11 (2.44)	17.86 (2.08)
Average physically unhealthy days/month	3.36 (0.37)	4.70 (0.52)	3.61 (0.43)	4.03 (0.33)
Percent smoking	14.04 (2.12)	20.67 (2.99)	15.70 (1.81)	17.93 (2.01)
Percent obesity	27.92 (4.90)	35.94 (5.03)	32.06 (4.37)	33.85 (3.93)
Percent physically inactive	20.61 (3.88)	31.86 (4.86)	25.80 (4.02)	27.72 (3.68)
Cardiovascular disease mortality rate	339.42 (54.33)	516.66 (91.38)	397.78 (66.65)	432.29 (57.85)
Cancer mortality rate	157.83 (20.17)	202.21 (31.76)	177.72 (26.85)	185.79 (18.50)
**Healthcare Access**				
Percent uninsured	8.15 (3.63)	13.20 (4.77)	10.36 (5.11)	10.72 (4.33)
Primary care physician rate	79.86 (41.33)	41.38 (23.25)	56.43 (28.58)	54.56 (28.65)
Preventable hospitalization rate	3912.93 (1238.16)	5983.81 (1953.21)	4081.10 (1573.13)	4991.30 (1028.37)
Percent vaccinated	50.18 (5.64)	38.93 (7.77)	40.28 (10.21)	46.88 (5.49)
Hospital beds per 100,000 persons	229.62 (290.35)	281.63 (385.68)	306.94 (455.30)	264.30 (275.85)
**Environmental and other**				
Social association rate	9.81 (3.78)	10.73 (4.17)	14.07 (5.72)	10.96 (3.55)
Average daily PM2.5	8.98 (1.95)	9.73 (1.33)	8.06 (1.81)	10.42 (1.35)

All values are mean (standard deviation) except for rural-urban continuum codes that is *N* (%). PM represents particulate matter

**Fig. 1:**
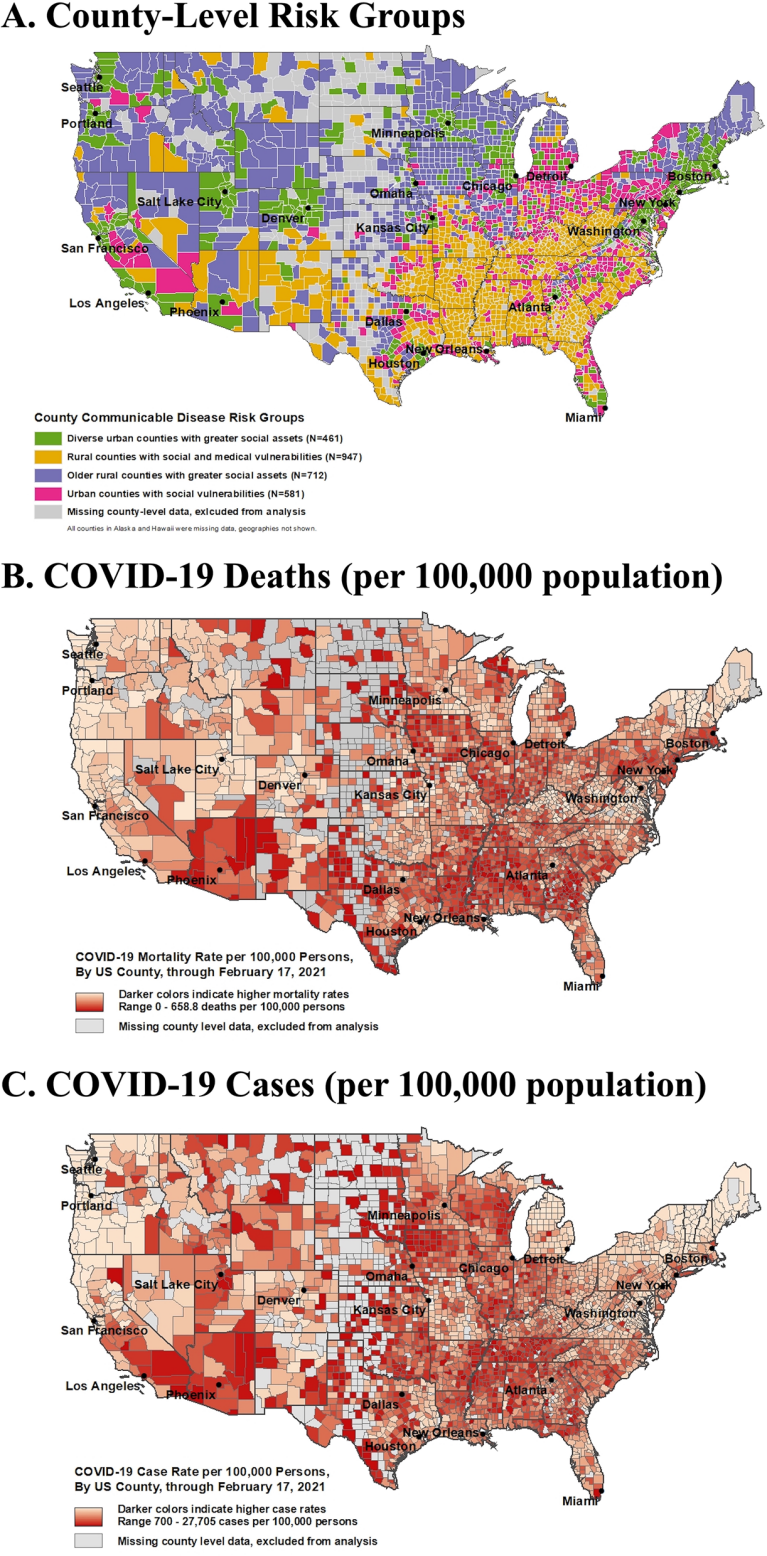
Nationwide Distribution of (**A**) County-Level Risk Groups (**B**) COVID-19 deaths per 100,000 and (**C**) COVID-19 cases per 100,00 from 1/21/20 to 2/17/21

Individual county-level metrics included in the latent class modeling were highly correlated with each other (Supplemental Figure 2). For example, favorable social and health assets tracked in counties with lower poverty metrics and higher median income; these counties also had more favorable health metrics such as proportions of smokers, people with obesity, and physically inactive people, as well as fewer years of potential life lost. Conversely, adverse social and health factors and a higher proportion of minority residents were observed in vulnerable counties. These counties also had, on average, lower median income, greater proportion of households with overcrowding, and poorer air pollution rates. Counties within the four groups have widespread geographic dispersion with some regional clustering (Figure 1A). For example, rural counties with social and health vulnerabilities are predominantly, but not exclusively, localized in the southern US.

### Nationwide COVID-19 Deaths and Cases

As of February 17^th^, 2021, 2,621 counties had reported at least 1 confirmed COVID-19 death and 2,701 counties had at least 1 confirmed COVID-19 case to be included in the publicly available dataset hosted by the NY Times. A total of 481,238 COVID-19 deaths and 25.9 million COVID-19 cases were included from 2/29/20 to 2/17/21 in 2701 counties in the US. Large between-county differences were evident with widespread geographic dispersion, with a significant gap in cumulative COVID-19 cases and death rates between counties in the 90^th^ and 10^th^ percentile of 6,581 and 291 per 100,000, respectively. Across US census regions, counties in the Midwest had the greatest cumulative rate of COVID-19 cases (8825) and counties in the Northeast had the greatest cumulative rate of COVID-19 deaths (190) (Figures 1B and C). Yet, there was wide geographic dispersion with counties with high burden of COVID-19 distributed across the US. Qualitatively, counties with high COVID-19 cases also had high COVID-19 deaths.

### Association Between County-Level Risk Groups and COVID-19 Outcomes

**Fig. 2:**
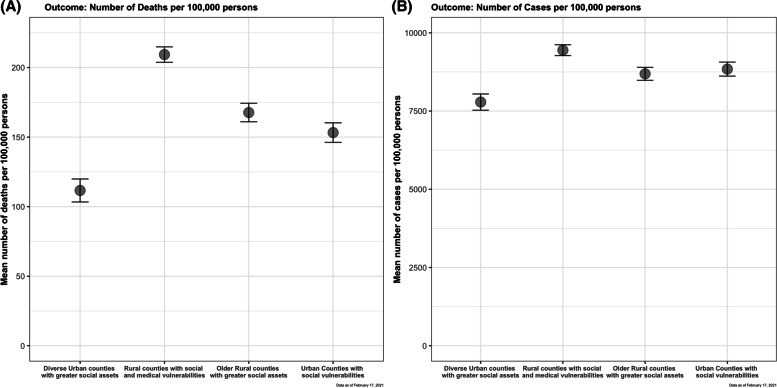
Association between county-level risk group and cumulative COVID-19 (**A**) deaths and (**B**) cases per 100,000 population from 1/21/20 to 2/17/21*

A significant association was observed between county-level risk groups and COVID-19 deaths and cases per 100,000, even after adjusting for time since first death and case, respectively, within each county (Figure 2, Table 2, Supplemental Table 5). Highest rates of COVID-19 cases (9557 [2520]) and deaths (210 [97]) per 100,000 population occurred in the cluster comprised of rural disadvantaged counties (p<0.05). Rates of COVID-19 deaths were nearly 2-fold higher in the rural disadvantaged cluster compared with the diverse urban counties with greater social assets.

**Table 2:** Summary of Covid-19 cases and deaths stratified by county-level risk groups from January 21^st^ 2020 to February 17^th^, 2021

	Diverse Urban counties with greater social assets	Rural Counties with social and medical vulnerabilities	Older Rural counties with greater social assets	Urban Counties with social vulnerabilities
Counties, N	448	916	677	580
Covid-19 related cases, N	13,906,385	3,383,189	1,4092,18	8,606,695
Covid-19 related cases per 100,000 persons, Mean (SD)	7803 (2614.3)	9556.6 (2519.6)	8771.7 (3121.9)	8825.8 (2275.6)
Covid-19 related deaths, N	238,268	72,412	25,639	144,919
Covid-19 related deaths per 100,000 persons, Mean (SD)	109 (59)	210 (97.2)	169.6 (98.1)	152 (61.3)

**Fig. 3:**
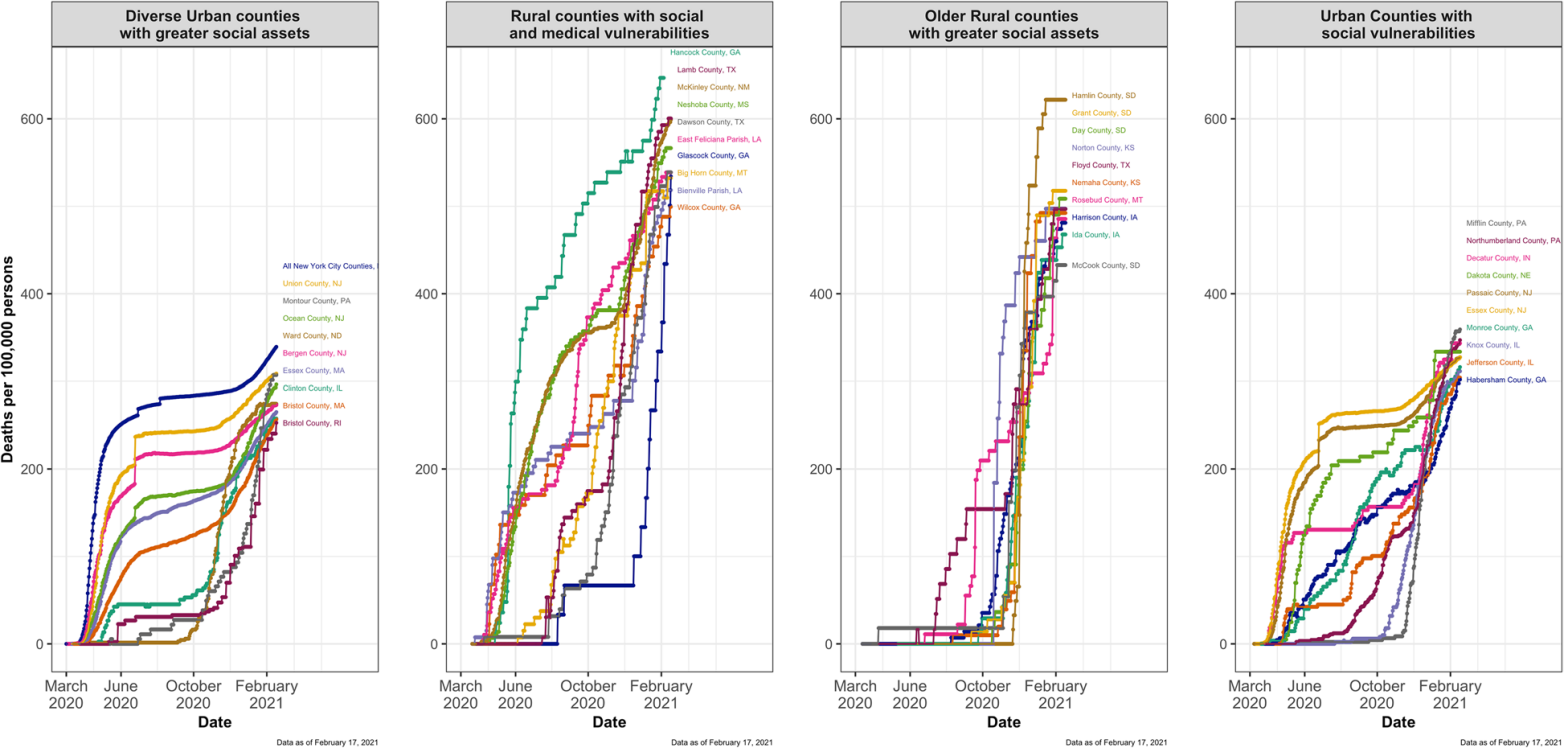
Spatiotemporal patterns of COVID-19 spread in the ten counties with the highest cumulative COVID-19 deaths per 100,000 from 1/21/20 to 2/17/21 within each county-level risk grouping

Within each county-level risk group, we next identified the ten counties with the highest cumulative COVID-19 deaths per 100,00 population and visualized the trajectory of COVID-19 mortality as a function of time since the 5^th^ reported COVID-19 death in each county (Figure 3). Hancock county, Georgia, in the vulnerable rural county-level risk group experienced the highest burden of COVID-19 deaths (622 per 100,000) while Norton County, Kansas, in the older rural county-level risk group had the highest level of cases (22,357 per 100,000) across the US (Supplemental Table 6). Yet, both county-level risk groups that predominantly included rural counties had the lowest rate of vaccination per 100,00 population compared with the two county-level risk groups predominantly comprised of urban counties (most data available as of 2/23/21). Within and across groups, there was no correlation between COVID-19 case and death rate and vaccination rate (Supplemental Figure 3).

## Discussion

This study describes a novel, data-driven, classification scheme that identified four distinct county-level risk groups, which were significantly associated with COVID-19 deaths and cases per 100,000 population from January 21, 2020 to February 17, 2021. The county-level risk group clustered by rural setting and social vulnerabilities had the greatest burden of COVID-19 deaths by nearly 2-fold, but the lowest documented rate of vaccination, to-date. This approach identified county-level vulnerability to COVID-19 deaths and cases by integrating characteristics across multiple key domains (sociodemographic, health-related, and environmental) implicated in the risk for COVID-19 infection and severity.

On a population level, targeting prevention of COVID-19 through vaccination, especially with resurgence in areas with novel variants, as well as directing resources to address long-term sequelae or disability due to COVID-19 infection requires identification of communities who have experienced greatest burden of COVID-19 infection and mortality [20, 21]. While there may be multiple mechanisms by which county-level factors may influence COVID-19 outcomes, classifying counties with similar socioecological profiles, which are implicated in COVID-19 infection rate and illness severity, will provide the ability for public health responses targeted to the communities with the greatest need. This is especially important at this stage in the pandemic when novel variants are emerging that may be more infectious or be associated with greater infection severity [22]. Other tools, such as the social vulnerability index (SVI) have been applied and demonstrated significant association between greater social vulnerability and both COVID-19 and non-COVID-19 health outcomes [23]. However, the SVI [24] relies only on demographic variables from the US census and does not integrate health status and healthcare access for county’s residents or environmental measures at the county-level, such as air pollution. Therefore, this current study extends prior reports by developing a comprehensive and data-driven classification scheme to reflect social, health, and environmental vulnerability at the county-level.

These data build upon prior studies that have documented substantial county-level disparities in COVID-19 as well as a large body of literature demonstrating similar patterns in geographic disparities across the US in non-communicable diseases, such as cardiovascular disease [25, 26]. Indeed, COVID-19 infection susceptibility and mortality has further amplified pre-existing place-based health disadvantage due to interrelated sociodemographic [27], health [28], and environmental factors [29] at the county-level and expand growing awareness of a “rural mortality penalty” [30] in the US. Recent investigations have demonstrated widening disparities in rural mortality rates due to noncommunicable diseases, such as chronic lung disease [31] and cardiovascular disease [32], compared with urban mortality rates. This likely reflects convergence of multiple ecological factors related to social determinants of overall health, such as economic prosperity [33] and education [27, 34]. Vulnerability as a result of underlying comorbidities may be one important factor in the observed place-based disparities, but does not account for intrinsic systemic and structural racism within and outside our healthcare system that may also be contributing [35]. Our study incorporates both sociodemographic factors and burden of chronic diseases at the county-level and provides a means to estimate susceptibility and burden of COVID-19 at the population level in hopes of mitigating avoidable health inequalities, especially in counties with limited resources.

Strengths of our study include use of innovative statistical methods to integrate 30 county-level characteristics that may be implicated in COVID-19 transmission and mortality with a latent-class modeling approach [18] that identified groups of counties with similar profiles of ecological risk factors. To accomplish this task, we harmonized and linked numerous publicly available county-level datasets to create a comprehensive classification scheme leveraged available data on county-level COVID-19 outcomes that represent cumulative cases and deaths from >12 months since the beginning of the pandemic.

This analysis has several limitations. First, there is the potential misclassification of COVID-19 cases and mortality due to under-ascertainment of COVID-19. Under-recognition of COVID-19 may have occurred due to limited availability of testing, especially earlier in the pandemic. Prior studies have demonstrated a strong correlation between testing and cases per 100,000 population and seroprevalence studies suggest that up to 10 times more COVID-19 infections occurred than number of cases [36, 37], which may reflect mild illness or no symptoms in addition to under-testing. Therefore, COVID-19 cases and deaths are unlikely to reflect the total burden of COVID-19. This has been demonstrated in analyses of excess deaths at the national level [38]. However, our analysis relies on the most comprehensive estimates of cumulative COVID-19 mortality and cases available at the county-level. Second, use of LCA assigns group membership for each county based on the highest probability, but there may be counties that may be equally likely to belong to another county-level risk group. However, examination of posterior probabilities revealed that no county had a probability <0.7 based on their assigned group membership and LCA facilitated the integration of 30 highly interrelated variables in one classification scheme in an unbiased data-driven approach. Third, our analysis does not account for individual-level characteristics that are associated with COVID-19 infection or disease severity. However, the current study design is ecological, and it is not meant to infer causation; rather it is meant to inform large-scale comparisons at the population-level to communicate with local health departments and leaders to inform decisions at the county-level [39]. Fourth, analysis at a smaller geographic scale, such as the neighborhood (census-tract) level was not possible in this nationwide study. Prior work in New York City [40], Milwaukee [41], and Chicago [42] have identified the neighborhood-level variation within a city in COVID-19 deaths that highlight structural inequities as root causes of these disparities and warrant further investigation. This analysis at the county-level is a meaningful geographic scale for changes in policy and public health response at the state- and national-level that can complement efforts within counties.

In conclusion, this study identified clustering of counties based on a variety of county-level factors that incorporate sociodemographic, health status and access, and environmental characteristics. The county-level risk index was significantly associated with COVID-19 cases and deaths, with the greatest burden for both occurring in the group with rural disadvantaged counties. Place-based health disadvantage for COVID-19 outcomes will require targeted and equitable allocation of resources for vaccination as well as management of long-term sequelae and disability due to COVID-19. The findings from this current study provide unique insights into county-level characteristics that may inform the lack of reserve or resilience at a population-level when challenged by an unexpected stress of a highly infectious communicable disease that carries a significant risk of morbidity and mortality, such as COVID-19.

## Supplementary Information

**Additional file 1.**

## Data Availability

The datasets used and/or analyzed during the current study are available in the New York Times GitHub repository (https://github.com/nytimes/COVID-19-data). Additional datasets generated for analysis are included in this published article and its supplementary information files.
