# Supplementary material for: Association between county-level risk groups and COVID-19 outcomes in the United States: a socioecological study
Source: BMC Public Health. 2022 Jan 13;22:81. doi: 10.1186/s12889-021-12469-y (PMC8756413; doi:10.1186/s12889-021-12469-y)
Supplement: Supplementary file 1 — Additional file 1. [file 12889_2021_12469_MOESM1_ESM.docx]

**ONLINE ONLY SUPPLEMENTAL FIGURES AND TABLES**

**Association Between County-Level Risk Groups and COVID-19 Outcomes in the United States: A Socioecological Study**

Sadiya S. Khan, MD, MSc; Amy E. Krefman, MS; Megan E. McCabe, MS; Lucia C. Petito, PhD; Xiaoyun Yang; Kiarri N. Kershaw, PhD; Lindsay R. Pool, PhD; Norrina B. Allen, PhD

**Supplemental Figure 1.** Demographic, Socioeconomic, Health Status, Healthcare Access, and Environmental Characteristics of Communicable Disease Risk (CDR) Groups (N=2701 counties in the United States)


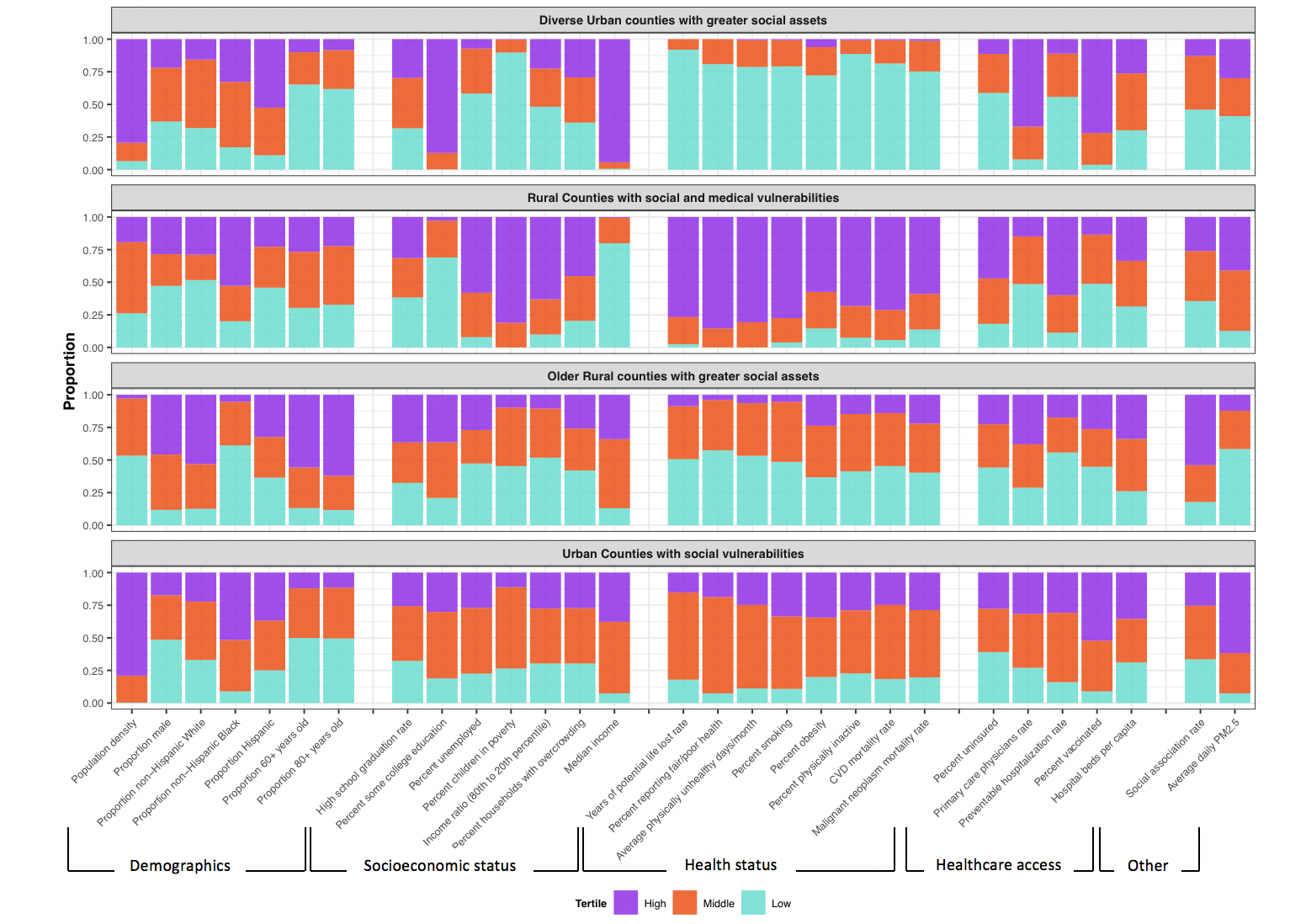


**Supplemental Figure 2.** Pearson correlation coefficients of county-level characteristics across multiple domains (demographics, socioeconomic status, health status, healthcare access, and other environmental factors) included in data-derived county-level risk groupings


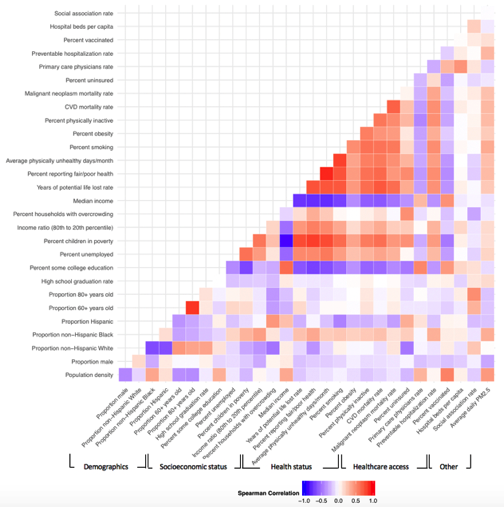


**Supplemental Figure 3. Correlation between COVID-19 cases, deaths, and vaccinations at the county-level in the top 40 counties overall and top 10 counties within county-level risk groups.**

**SUPPLEMENTAL TABLES**

**Supplemental Table 1.** Data sources, file, and year used for county-level characteristics across multiple domains included in Communicable Disease Risk Group classification

| **County-level factor^1^** | **Source** | **File** | **Year used** |
| --- | --- | --- | --- |
| **Demographic characteristics of residents** |  |  |  |
| Age $\geq$60 years | U.S. Census Bureau | County Characteristics Resident Population Estimates | 2018 |
| Age $\geq$80 years | U.S. Census Bureau | County Characteristics Resident Population Estimates | 2018 |
| Male | U.S. Census Bureau | County Characteristics Resident Population Estimates | 2018 |
| Non-Hispanic White alone | U.S. Census Bureau | County Characteristics Resident Population Estimates |  |
| Non-Hispanic Black alone | U.S. Census Bureau | County Characteristics Resident Population Estimates | 2018 |
| Hispanic | U.S. Census Bureau | County Characteristics Resident Population Estimates | 2018 |
| Population density, people per square mile | U.S. Census Bureau | Population, Housing Units, Area, and Density/County Characteristics Resident Population Estimates | 2010/2018^2^ |
| Rural Urban Continuum Codes, categories | U.S. Department of Agriculture | 2013 Rural Urban Continuum Codes | 2013 |
| **Socioeconomic characteristics of residents** |  |  |  |
| High school graduation, rate of graduation among those expected to graduate | Robert Wood Johnson Foundation, County Health Rankings | County Health Rankings and Roadmaps: 2020 State Report Data | 2016-2017 |
| Some college education, percentage of adults age 25-44 with some post-secondary education | U.S. Census Bureau | American Community Survey | 2014-2018 |
| Unemployment, percentage of population ages 16+ unemployed and looking for work | Bureau of Labor Statistics | Local Area Unemployment Statistics | 2018 |
| Children in poverty, percentage of children (under age 18) living in poverty | U.S. Census Bureau | Small Area Income and Poverty Estimates | 2018 |
| Income inequality, ratio of household income at the 80th percentile to income at the 20th percentile | U.S. Census Bureau | American Community Survey | 2014-2018 |
| Overcrowding, percentage of households with overcrowding | U.S. Department of Housing and Urban Development | Comprehensive Housing Affordability Strategy (CHAS) data | 2012-2016 |
| Income, median household income in 2018 inflation-adjusted dollars | U.S. Census Bureau | Small Area Income and Poverty Estimates | 2018 |
| **Health status of residents, %** |  |  |  |
| Years of potential life lost, age-adjusted rate per 100,000 | Robert Wood Johnson Foundation, County Health Rankings | National Center for Health Statistics-Mortality Files | 2016-2018 |
| Fair or poor health, percentage of adults that report fair or poor health | Robert Wood Johnson Foundation, County Health Rankings | Behavior Risk Factor Surveillance System | 2017 |
| Physically unhealthy days, average number of days per month | Robert Wood Johnson Foundation, County Health Rankings | Behavior Risk Factor Surveillance System | 2017 |
| Smoking, percentage adults reporting current smoking | Robert Wood Johnson Foundation, County Health Rankings | Behavior Risk Factor Surveillance System | 2017 |
| Obesity, percentage adults reporting BMI ≥30kg/m^2^ | Robert Wood Johnson Foundation, County Health Rankings | United States Diabetes Surveillance System | 2016 |
| Physical inactivity, percentage of adults that report no leisure-time physical activity | Robert Wood Johnson Foundation, County Health Rankings | United States Diabetes Surveillance System | 2016 |
| CVD mortality, age-adjusted rate | Centers for Disease Control and Prevention | CDC WONDER | 2016-2018 |
| Malignant neoplasm mortality, age-adjusted rate | Centers for Disease Control and Prevention | CDC WONDER | 2016-2018 |
| **Healthcare access characteristics** |  |  |  |
| Uninsured, percentage of people under age 65 without insurance | U.S. Census Bureau | Small Area Health Insurance Estimates Program | 2017 |
| Primary care physicians, rate per 100,000 | U.S. Census Bureau | Small Area Health Insurance Estimates Program, American Medical Association | 2017 |
| Preventable hospitalization rate, discharges for ambulatory care sensitive conditions per 100,000 Medicare enrollees | Centers for Medicare and Medicaid | Mapping Medicare Disparities Tool | 2017 |
| Percent vaccinated, Percentage of annual Medicare enrollees having an annual flu vaccination | Centers for Medicare and Medicaid | Mapping Medicare Disparities Tool | 2017 |
| Hospital beds, number^3^ | Health Resources and Services Administration | Area Health Resources Files | 2018-2019 |
| **Other** |  |  |  |
| Social association rate, associations per 10,000 population | Robert Wood Johnson Foundation, County Health Rankings | County Health Rankings and Roadmaps: 2020 State Report Data | 2017 |
| PM2.5, average daily amount of fine particulate matter in micrograms per cubic meter | Centers for Disease Control | Environmental Public Health Tracking Network | 2014 |
|  |  |  |  |

^1^ All measures are percentages or proportions of the total population unless indicated otherwise.

^2^ Land area estimates from 2010 and total county population estimates from 2018 were used to calculate an estimated population density for 2018.

^3^ Using 2018 population totals for the county and the number of hospital beds, calculated hospital beds per capita.

^4^Most recent publicly available versions of each respective data source was utilized

**Supplemental Table 2.** Latent class analysis model fit statistics for latent classes considered for Communicable Disease Risk Group classification

| Model | log-likelihood | Residual DF | BIC | aBIC | cAIC | likelihood-ratio |
| --- | --- | --- | --- | --- | --- | --- |
| Model 1 | -91436.62 | 2635 | 183394.7 | 183185.0 | 183460.7 | 140195.5 |
| Model 2 | -83506.38 | 2568 | 168063.6 | 167641.1 | 168196.6 | 124335.1 |
| Model 3 | -81207.10 | 2501 | 163994.5 | 163359.0 | 164194.5 | 119736.5 |
| Model 4 | **-79427.57** | **2434** | **160964.8** | **160116.5** | **161231.8** | **116177.4** |
| Model 5 | -78350.41 | 2367 | 159339.9 | 158278.7 | 159673.9 | 114023.1 |

**Supplemental Table 3.** Probability of responses to county metrics for 4 class model, among counties used in latent class analysis for Communicable Disease Risk Group classification

| County-level Variable |  | Diverse Urban counties with greater social assets | Rural counties with social and medical vulnerabilities | Older Rural counties with greater social assets | Urban Counties with social vulnerabilities |
| --- | --- | --- | --- | --- | --- |
| Population density (%) |  |  |  |  |  |
|  | Pr(1) | 0.26 | 0.54 | 0.07 | 0.01 |
|  | Pr(2) | 0.55 | 0.44 | 0.14 | 0.21 |
|  | Pr(3) | 0.19 | 0.03 | 0.79 | 0.78 |
| Proportion male (%) |  |  |  |  |  |
|  | Pr(1) | 0.47 | 0.12 | 0.37 | 0.49 |
|  | Pr(2) | 0.24 | 0.42 | 0.41 | 0.34 |
|  | Pr(3) | 0.29 | 0.46 | 0.22 | 0.17 |
| Proportion non-Hispanic White (%) |  |  |  |  |  |
|  | Pr(1) | 0.52 | 0.13 | 0.31 | 0.33 |
|  | Pr(2) | 0.20 | 0.34 | 0.53 | 0.45 |
|  | Pr(3) | 0.29 | 0.53 | 0.16 | 0.22 |
| Proportion non-Hispanic Black (%) |  |  |  |  |  |
|  | Pr(1) | 0.20 | 0.61 | 0.17 | 0.09 |
|  | Pr(2) | 0.27 | 0.33 | 0.50 | 0.40 |
|  | Pr(3) | 0.53 | 0.05 | 0.33 | 0.51 |
| Proportion Hispanic (%) |  |  |  |  |  |
|  | Pr(1) | 0.46 | 0.37 | 0.11 | 0.25 |
|  | Pr(2) | 0.31 | 0.31 | 0.37 | 0.38 |
|  | Pr(3) | 0.23 | 0.32 | 0.52 | 0.37 |
| Proportion 60+ years old (%) |  |  |  |  |  |
|  | Pr(1) | 0.30 | 0.13 | 0.65 | 0.50 |
|  | Pr(2) | 0.43 | 0.31 | 0.25 | 0.38 |
|  | Pr(3) | 0.27 | 0.56 | 0.10 | 0.12 |
| Proportion 80+ years old (%) |  |  |  |  |  |
|  | Pr(1) | 0.33 | 0.12 | 0.62 | 0.49 |
|  | Pr(2) | 0.45 | 0.26 | 0.30 | 0.39 |
|  | Pr(3) | 0.22 | 0.62 | 0.09 | 0.12 |
| Rural-Urban Continuum Code (%) |  |  |  |  |  |
|  | Pr(1) | 0.038 | 0.013 | 0.451 | 0.265 |
|  | Pr(2) | 0.091 | 0.041 | 0.214 | 0.258 |
|  | Pr(3) | 0.087 | 0.070 | 0.165 | 0.207 |
|  | Pr(4) | 0.073 | 0.047 | 0.070 | 0.133 |
|  | Pr(5) | 0.036 | 0.038 | 0.031 | 0.024 |
|  | Pr(6) | 0.320 | 0.293 | 0.025 | 0.099 |
|  | Pr(7) | 0.168 | 0.320 | 0.031 | 0.009 |
|  | Pr(8) | 0.087 | 0.079 | 0.004 | 0.004 |
|  | Pr(9) | 0.101 | 0.100 | 0.007 | 0.000 |
| High school graduation rate (%) |  |  |  |  |  |
|  | Pr(1) | 0.38 | 0.32 | 0.32 | 0.33 |
|  | Pr(2) | 0.30 | 0.31 | 0.39 | 0.41 |
|  | Pr(3) | 0.32 | 0.36 | 0.29 | 0.26 |
| Percent some college education (%) |  |  |  |  |  |
|  | Pr(1) | 0.69 | 0.21 | 0.00 | 0.19 |
|  | Pr(2) | 0.28 | 0.43 | 0.13 | 0.51 |
|  | Pr(3) | 0.03 | 0.36 | 0.87 | 0.30 |
| Percent unemployed (%) |  |  |  |  |  |
|  | Pr(1) | 0.08 | 0.47 | 0.59 | 0.22 |
|  | Pr(2) | 0.34 | 0.26 | 0.34 | 0.50 |
|  | Pr(3) | 0.58 | 0.27 | 0.07 | 0.27 |
| Percent children in poverty (%) |  |  |  |  |  |
|  | Pr(1) | 0.00 | 0.45 | 0.89 | 0.27 |
|  | Pr(2) | 0.19 | 0.45 | 0.10 | 0.61 |
|  | Pr(3) | 0.81 | 0.10 | 0.00 | 0.12 |
| Income ratio |  |  |  |  |  |
| (80^th^ to 20^th^ percentile) (%) | Pr(1) | 0.10 | 0.52 | 0.48 | 0.30 |
|  | Pr(2) | 0.27 | 0.38 | 0.29 | 0.42 |
|  | Pr(3) | 0.63 | 0.10 | 0.22 | 0.28 |
| Percent households with |  |  |  |  |  |
| overcrowding (%) | Pr(1) | 0.20 | 0.42 | 0.36 | 0.30 |
|  | Pr(2) | 0.34 | 0.32 | 0.35 | 0.42 |
|  | Pr(3) | 0.45 | 0.26 | 0.29 | 0.28 |
| Median income (%) |  |  |  |  |  |
|  | Pr(1) | 0.80 | 0.13 | 0.01 | 0.08 |
|  | Pr(2) | 0.19 | 0.53 | 0.05 | 0.55 |
|  | Pr(3) | 0.01 | 0.34 | 0.94 | 0.37 |
| Years of potential life lost rate (%) |  |  |  |  |  |
|  | Pr(1) | 0.03 | 0.51 | 0.92 | 0.18 |
|  | Pr(2) | 0.21 | 0.40 | 0.08 | 0.66 |
|  | Pr(3) | 0.77 | 0.09 | 0.00 | 0.16 |
| Percent reporting fair/poor |  |  |  |  |  |
| health (%) | Pr(1) | 0.00 | 0.57 | 0.81 | 0.07 |
|  | Pr(2) | 0.15 | 0.38 | 0.19 | 0.73 |
|  | Pr(3) | 0.85 | 0.04 | 0.00 | 0.20 |
| Average physically unhealthy |  |  |  |  |  |
| days per month (%) | Pr(1) | 0.00 | 0.53 | 0.79 | 0.11 |
|  | Pr(2) | 0.19 | 0.40 | 0.20 | 0.64 |
|  | Pr(3) | 0.81 | 0.07 | 0.01 | 0.25 |
| Percent smoking (%) |  |  |  |  |  |
|  | Pr(1) | 0.04 | 0.49 | 0.78 | 0.11 |
|  | Pr(2) | 0.19 | 0.46 | 0.21 | 0.55 |
|  | Pr(3) | 0.77 | 0.06 | 0.01 | 0.34 |
| Percent obesity (%) |  |  |  |  |  |
|  | Pr(1) | 0.14 | 0.37 | 0.72 | 0.21 |
|  | Pr(2) | 0.28 | 0.39 | 0.22 | 0.45 |
|  | Pr(3) | 0.57 | 0.24 | 0.06 | 0.34 |
| Percent physically inactive (%) |  |  |  |  |  |
|  | Pr(1) | 0.08 | 0.41 | 0.88 | 0.23 |
|  | Pr(2) | 0.24 | 0.44 | 0.11 | 0.48 |
|  | Pr(3) | 0.68 | 0.15 | 0.01 | 0.29 |
| Cardiovascular disease |  |  |  |  |  |
| mortality rate (%) | Pr(1) | 0.06 | 0.46 | 0.81 | 0.19 |
|  | Pr(2) | 0.23 | 0.40 | 0.19 | 0.56 |
|  | Pr(3) | 0.71 | 0.14 | 0.01 | 0.25 |
| Cancer mortality rate (%) |  |  |  |  |  |
|  | Pr(1) | 0.14 | 0.41 | 0.74 | 0.20 |
|  | Pr(2) | 0.27 | 0.38 | 0.24 | 0.51 |
|  | Pr(3) | 0.59 | 0.22 | 0.02 | 0.29 |
| Percent uninsured (%) |  |  |  |  |  |
|  | Pr(1) | 0.18 | 0.45 | 0.59 | 0.39 |
|  | Pr(2) | 0.35 | 0.33 | 0.30 | 0.33 |
|  | Pr(3) | 0.47 | 0.22 | 0.11 | 0.28 |
| Primary care physician rate (%) |  |  |  |  |  |
|  | Pr(1) | 0.49 | 0.29 | 0.08 | 0.27 |
|  | Pr(2) | 0.36 | 0.33 | 0.25 | 0.41 |
|  | Pr(3) | 0.15 | 0.38 | 0.67 | 0.32 |
| Preventible hospitalization rate (%) |  |  |  |  |  |
|  | Pr(1) | 0.11 | 0.56 | 0.56 | 0.16 |
|  | Pr(2) | 0.29 | 0.27 | 0.34 | 0.52 |
|  | Pr(3) | 0.60 | 0.17 | 0.11 | 0.31 |
| Percent vaccinated (%) |  |  |  |  |  |
|  | Pr(1) | 0.49 | 0.45 | 0.04 | 0.09 |
|  | Pr(2) | 0.38 | 0.29 | 0.25 | 0.39 |
|  | Pr(3) | 0.13 | 0.26 | 0.71 | 0.52 |
| Hospital beds per capita (%) |  |  |  |  |  |
|  | Pr(1) | 0.31 | 0.26 | 0.30 | 0.31 |
|  | Pr(2) | 0.35 | 0.40 | 0.44 | 0.33 |
|  | Pr(3) | 0.33 | 0.34 | 0.26 | 0.36 |
| Social association rate (%) |  |  |  |  |  |
|  | Pr(1) | 0.35 | 0.18 | 0.46 | 0.33 |
|  | Pr(2) | 0.39 | 0.28 | 0.41 | 0.41 |
|  | Pr(3) | 0.26 | 0.54 | 0.13 | 0.26 |
| Average daily PM2.5 (%) |  |  |  |  |  |
|  | Pr(1) | 0.13 | 0.58 | 0.41 | 0.08 |
|  | Pr(2) | 0.46 | 0.30 | 0.29 | 0.31 |
|  | Pr(3) | 0.41 | 0.12 | 0.30 | 0.61 |

**Supplemental Table 4.** Classification of county metrics for 4 class model, among counties used in latent class analysis for Communicable Disease Risk Group classification

|  | **level** | **Diverse Urban counties with greater social assets** | **Rural counties with social and medical vulnerabilities** | **Older Rural counties with greater social assets** | **Urban Counties with social vulnerabilities** | **p-value** |
| --- | --- | --- | --- | --- | --- | --- |
| n |  | 461 | 947 | 712 | 581 |  |
| Population density (%) | (24.4,79.3] | 65 (14.1) | 516 (54.5) | 312 (43.8) | 120 (20.7) | <0.001 |
|  | (79.3,3.7e+04] | 366 (79.4) | 183 (19.3) | 20 ( 2.8) | 459 (79.0) |  |
|  | [0.0366,24.4] | 30 ( 6.5) | 248 (26.2) | 380 (53.4) | 2 ( 0.3) |  |
| Proportion male (%) | (0.493,0.502] | 190 (41.2) | 230 (24.3) | 302 (42.4) | 198 (34.1) | <0.001 |
|  | (0.502,0.732] | 101 (21.9) | 270 (28.5) | 326 (45.8) | 101 (17.4) |  |
|  | [0.431,0.493] | 170 (36.9) | 447 (47.2) | 84 (11.8) | 282 (48.5) |  |
| Proportion non-Hispanic White (%) | (0.716,0.9] | 242 (52.5) | 184 (19.4) | 244 (34.3) | 260 (44.8) | <0.001 |
|  | (0.9,0.979] | 72 (15.6) | 274 (28.9) | 378 (53.1) | 129 (22.2) |  |
|  | [0.0269,0.716] | 147 (31.9) | 489 (51.6) | 90 (12.6) | 192 (33.0) |  |
| Proportion non-Hispanic Black (%) | (0.0116,0.068] | 231 (50.1) | 258 (27.2) | 238 (33.4) | 229 (39.4) | <0.001 |
|  | (0.068,0.824] | 151 (32.8) | 499 (52.7) | 38 ( 5.3) | 300 (51.6) |  |
|  | [0,0.0116] | 79 (17.1) | 190 (20.1) | 436 (61.2) | 52 ( 9.0) |  |
| Proportion Hispanic (%) | (0.029,0.0707] | 168 (36.4) | 297 (31.4) | 222 (31.2) | 222 (38.2) | <0.001 |
|  | (0.0707,0.964] | 242 (52.5) | 216 (22.8) | 230 (32.3) | 213 (36.7) |  |
|  | [0.0061,0.029] | 51 (11.1) | 434 (45.8) | 260 (36.5) | 146 (25.1) |  |
| Proportion 60+ years old (%) | (0.239,0.281] | 114 (24.7) | 407 (43.0) | 221 (31.0) | 221 (38.0) | <0.001 |
|  | (0.281,0.664] | 46 (10.0) | 253 (26.7) | 397 (55.8) | 70 (12.0) |  |
|  | [0.069,0.239] | 301 (65.3) | 287 (30.3) | 94 (13.2) | 290 (49.9) |  |
| Proportion 80+ years old (%) | (0.0403,0.0512] | 137 (29.7) | 426 (45.0) | 187 (26.3) | 226 (38.9) | <0.001 |
|  | (0.0512,0.227] | 39 ( 8.5) | 211 (22.3) | 442 (62.1) | 67 (11.5) |  |
|  | [0,0.0403] | 285 (61.8) | 310 (32.7) | 83 (11.7) | 288 (49.6) |  |
| Rural-Urban Continuum Code (%) | 1 | 212 (46.0) | 36 ( 3.8) | 8 ( 1.1) | 155 (26.7) | <0.001 |
|  | 2 | 100 (21.7) | 86 ( 9.1) | 28 ( 3.9) | 152 (26.2) |  |
|  | 3 | 74 (16.1) | 82 ( 8.7) | 52 ( 7.3) | 122 (21.0) |  |
|  | 4 | 31 ( 6.7) | 72 ( 7.6) | 33 ( 4.6) | 77 (13.3) |  |
|  | 5 | 14 ( 3.0) | 35 ( 3.7) | 28 ( 3.9) | 12 ( 2.1) |  |
|  | 6 | 10 ( 2.2) | 301 (31.8) | 210 (29.5) | 57 ( 9.8) |  |
|  | 7 | 15 ( 3.3) | 158 (16.7) | 225 (31.6) | 5 ( 0.9) |  |
|  | 8 | 2 ( 0.4) | 83 ( 8.8) | 56 ( 7.9) | 1 ( 0.2) |  |
|  | 9 | 3 ( 0.7) | 94 ( 9.9) | 72 (10.1) | 0 ( 0.0) |  |
| High school graduation rate (%) | (87,92.4] | 178 (38.6) | 286 (30.2) | 221 (31.0) | 244 (42.0) | <0.001 |
|  | (92.4,100] | 137 (29.7) | 298 (31.5) | 260 (36.5) | 149 (25.6) |  |
|  | [25.6,87] | 146 (31.7) | 363 (38.3) | 231 (32.4) | 188 (32.4) |  |
| Percent some college education (%) | (52.6,63.4] | 58 (12.6) | 267 (28.2) | 304 (42.7) | 296 (50.9) | <0.001 |
|  | (63.4,100] | 402 (87.2) | 27 ( 2.9) | 259 (36.4) | 175 (30.1) |  |
|  | [15.2,52.6] | 1 ( 0.2) | 653 (69.0) | 149 (20.9) | 110 (18.9) |  |
| Percent unemployed (%) | (3.39,4.43] | 159 (34.5) | 323 (34.1) | 183 (25.7) | 293 (50.4) | <0.001 |
|  | (4.43,18.1] | 33 ( 7.2) | 550 (58.1) | 192 (27.0) | 157 (27.0) |  |
|  | [1.3,3.39] | 269 (58.4) | 74 ( 7.8) | 337 (47.3) | 131 (22.5) |  |
| Percent children in poverty (%) | (16.4,23.9] | 45 ( 9.8) | 179 (18.9) | 319 (44.8) | 362 (62.3) | <0.001 |
|  | (23.9,68.3] | 2 ( 0.4) | 768 (81.1) | 70 ( 9.8) | 65 (11.2) |  |
|  | [2.5,16.4] | 414 (89.8) | 0 ( 0.0) | 323 (45.4) | 154 (26.5) |  |
| Income ratio  (80^th^ to 20^th^ percentile) (%) | (4.14,4.67] | 135 (29.3) | 256 (27.0) | 268 (37.6) | 245 (42.2) | <0.001 |
|  | (4.67,12] | 104 (22.6) | 597 (63.0) | 75 (10.5) | 160 (27.5) |  |
|  | [2.54,4.14] | 222 (48.2) | 94 ( 9.9) | 369 (51.8) | 176 (30.3) |  |
| Percent households with  overcrowding (%) | (1.44,2.45] | 160 (34.7) | 325 (34.3) | 229 (32.2) | 247 (42.5) | <0.001 |
|  | (2.45,38.1] | 135 (29.3) | 429 (45.3) | 184 (25.8) | 158 (27.2) |  |
|  | [0,1.44] | 166 (36.0) | 193 (20.4) | 299 (42.0) | 176 (30.3) |  |
| Median income (%) | (4.51e+04,5.47e+04] | 23 ( 5.0) | 185 (19.5) | 377 (52.9) | 319 (54.9) | <0.001 |
|  | (5.47e+04,1.36e+05] | 435 (94.4) | 6 ( 0.6) | 242 (34.0) | 219 (37.7) |  |
|  | [2.02e+04,4.51e+04] | 3 ( 0.7) | 756 (79.8) | 93 (13.1) | 43 ( 7.4) |  |
| Years of potential life lost rate (%) | (7.27e+03,9.49e+03] | 37 ( 8.0) | 197 (20.8) | 289 (40.6) | 389 (67.0) | <0.001 |
|  | (9.49e+03,2.91e+04] | 0 ( 0.0) | 727 (76.8) | 62 ( 8.7) | 88 (15.1) |  |
|  | [2.73e+03,7.27e+03] | 424 (92.0) | 23 ( 2.4) | 361 (50.7) | 104 (17.9) |  |
| Percent reporting fair/poor  health (%) | (15.3,19.7] | 88 (19.1) | 138 (14.6) | 275 (38.6) | 429 (73.8) | <0.001 |
|  | (19.7,41] | 0 ( 0.0) | 809 (85.4) | 28 ( 3.9) | 109 (18.8) |  |
|  | [8.12,15.3] | 373 (80.9) | 0 ( 0.0) | 409 (57.4) | 43 ( 7.4) |  |
| Average physically unhealthy  days per month (%) | (3.64,4.24] | 95 (20.6) | 183 (19.3) | 287 (40.3) | 372 (64.0) | <0.001 |
|  | (4.24,6.55] | 3 ( 0.7) | 764 (80.7) | 46 ( 6.5) | 144 (24.8) |  |
|  | [2.45,3.64] | 363 (78.7) | 0 ( 0.0) | 379 (53.2) | 65 (11.2) |  |
| Percent smoking (%) | (15.5,18.7] | 93 (20.2) | 177 (18.7) | 327 (45.9) | 323 (55.6) | <0.001 |
|  | (18.7,41.5] | 3 ( 0.7) | 734 (77.5) | 39 ( 5.5) | 195 (33.6) |  |
|  | [5.91,15.5] | 365 (79.2) | 36 ( 3.8) | 346 (48.6) | 63 (10.8) |  |
| Percent obesity (%) | (30.7,35.2] | 100 (21.7) | 265 (28.0) | 281 (39.5) | 265 (45.6) | <0.001 |
|  | (35.2,57.7] | 28 ( 6.1) | 544 (57.4) | 169 (23.7) | 200 (34.4) |  |
|  | [12.4,30.7] | 333 (72.2) | 138 (14.6) | 262 (36.8) | 116 (20.0) |  |
| Percent physically inactive (%) | (25,29.7] | 49 (10.6) | 230 (24.3) | 313 (44.0) | 281 (48.4) | <0.001 |
|  | (29.7,49.9] | 3 ( 0.7) | 646 (68.2) | 105 (14.7) | 168 (28.9) |  |
|  | [9.5,25] | 409 (88.7) | 71 ( 7.5) | 294 (41.3) | 132 (22.7) |  |
| Cardiovascular disease  mortality rate (%) | (386,466] | 83 (18.0) | 218 (23.0) | 289 (40.6) | 330 (56.8) | <0.001 |
|  | (466,1.02e+03] | 3 ( 0.7) | 675 (71.3) | 100 (14.0) | 144 (24.8) |  |
|  | [87.8,386] | 375 (81.3) | 54 ( 5.7) | 323 (45.4) | 107 (18.4) |  |
| Cancer mortality rate (%) | (171,195] | 108 (23.4) | 258 (27.2) | 268 (37.6) | 300 (51.6) | <0.001 |
|  | (195,511] | 6 ( 1.3) | 559 (59.0) | 157 (22.1) | 167 (28.7) |  |
|  | [59.6,171] | 347 (75.3) | 130 (13.7) | 287 (40.3) | 114 (19.6) |  |
| Percent uninsured (%) | (12.9,33.7] | 52 (11.3) | 446 (47.1) | 161 (22.6) | 161 (27.7) | <0.001 |
|  | (8.33,12.9] | 138 (29.9) | 330 (34.8) | 236 (33.1) | 193 (33.2) |  |
|  | [2.26,8.33] | 271 (58.8) | 171 (18.1) | 315 (44.2) | 227 (39.1) |  |
| Primary care physician rate (%) | (37.2,62.2] | 116 (25.2) | 346 (36.5) | 237 (33.3) | 240 (41.3) | <0.001 |
|  | (62.2,514] | 309 (67.0) | 141 (14.9) | 270 (37.9) | 184 (31.7) |  |
|  | [0,37.2] | 36 ( 7.8) | 460 (48.6) | 205 (28.8) | 157 (27.0) |  |
| Preventible hospitalization rate (%) | (4e+03,5.39e+03] | 153 (33.2) | 272 (28.7) | 191 (26.8) | 309 (53.2) | <0.001 |
|  | (5.39e+03,1.69e+04] | 51 (11.1) | 568 (60.0) | 124 (17.4) | 179 (30.8) |  |
|  | [536,4e+03] | 257 (55.7) | 107 (11.3) | 397 (55.8) | 93 (16.0) |  |
| Percent vaccinated (%) | (39,47] | 112 (24.3) | 357 (37.7) | 206 (28.9) | 226 (38.9) | <0.001 |
|  | (47,66] | 332 (72.0) | 128 (13.5) | 187 (26.3) | 303 (52.2) |  |
|  | [4,39] | 17 ( 3.7) | 462 (48.8) | 319 (44.8) | 52 ( 9.0) |  |
| Hospital beds per capita (%) | (0.00113,0.00284] | 201 (43.6) | 332 (35.1) | 285 (40.0) | 194 (33.4) | 0.001 |
|  | (0.00284,0.131] | 121 (26.2) | 318 (33.6) | 241 (33.8) | 206 (35.5) |  |
|  | [0,0.00113] | 139 (30.2) | 297 (31.4) | 186 (26.1) | 181 (31.2) |  |
| Social association rate (%) | (13.3,52.3] | 59 (12.8) | 247 (26.1) | 384 (53.9) | 147 (25.3) | <0.001 |
|  | (9.27,13.3] | 190 (41.2) | 364 (38.4) | 201 (28.2) | 239 (41.1) |  |
|  | [0,9.27] | 212 (46.0) | 336 (35.5) | 127 (17.8) | 195 (33.6) |  |
| Average daily PM2.5 (%) | (10,19.7] | 138 (29.9) | 388 (41.0) | 87 (12.2) | 359 (61.8) | <0.001 |
|  | (8.5,10] | 134 (29.1) | 439 (46.4) | 209 (29.4) | 179 (30.8) |  |
|  | [3,8.5] | 189 (41.0) | 120 (12.7) | 416 (58.4) | 43 ( 7.4) |  |

**Supplemental Table 5.** Coefficients from adjusted linear regression models for primary and sensitivity analyses to evaluate the association between county-level risk groups and cumulative COVID-19 deaths and cases per 100,000 persons from January 21^st^ 2020 to February 17^th^ 2021

|  | **Deaths per 100,000** | **Cases per 100,000** | **Deaths per 100,000 (sensitivity)** |
| --- | --- | --- | --- |
| ***Number of counties included*** | 2621 | 2701 | 2694 |
| Intercept | 183.7* | 8858.6* | 88.9* |
| Diverse Urban counties with greater social assets | Ref. | Ref. | Ref. |
| Rural Counties with social and medical vulnerabilities | 97.7* | 1657.9* | 98.8* |
| Older Rural counties with greater social assets | 56.0* | 902.5* | 58.2* |
| Urban Counties with social vulnerabilities | 41.6* | 1053.6* | 45.4* |
| Time since 1^st^ case (days) | -0.22* | -3.29 | 0.05 |
| ***LRT for group term p-value*** | 4.40e-79 | 1.32e-23 | 1.39e-78 |

*Indicates a model coefficient which was significant at the p < 0.05 level

**Supplemental Table 6.** Quantification of COVID-19 burden (cases, deaths) and vaccination in the ten counties with the highest cumulative deaths per 100,000 persons from January 21^st^ 2020 to February 17^th^ 2021 within each county-level risk group

| County | State | Date of 1^st^ case | Date of 1^st^ death | Cases per 100,000 persons | Deaths per 100,000 persons | | Vaccination Rates per 100,000 persons | | | | | |
| --- | --- | --- | --- | --- | --- | --- | --- | --- | --- | --- | --- | --- |
|  |  |  |  |  |  | | 1^st^ dose | | 2^nd^ dose | | Data through | |
| *Diverse urban counties with greater social assets* | | | | | |  | |  | |  | |  |
| All New York City Counties | NY | 2020-03-01 | 2020-03-14 | 8080.2 | 339.3 | | 11472.4 | | 5522.3 | | 2/17/21 | |
| Union County | NJ | 2020-03-09 | 2020-03-23 | 9780.2 | 308.6 | | 10256.3 | | 4791.9 | | 2/24/21 | |
| Montour County | PA | 2020-03-21 | 2020-05-15 | 9490.1 | 307 | | 15164.5 | | 18059.2 | | 2/23/21 | |
| Ocean County | NJ | 2020-03-13 | 2020-03-24 | 9245.1 | 296.4 | | 12316.6 | | 5312.9 | | 2/24/21 | |
| Ward County | ND | 2020-03-11 | 2020-04-12 | 13385.7 | 274.6 | | 14400.0 | | 8400.0 | | 2/23/21 | |
| Bergen County | NJ | 2020-03-04 | 2020-03-10 | 7841.9 | 273.2 | | 14431.4 | | 6768.2 | | 2/24/21 | |
| Essex County | MA | 2020-03-10 | 2020-03-23 | 10319.9 | 265.1 | | 12800.0 | | 4100.0 | | 2/23/21 | |
| Clinton County | IL | 2020-03-15 | 2020-04-24 | 14684.2 | 257.7 | | 23690.9 | | 6974.1 | |  | |
| Bristol County | MA | 2020-03-14 | 2020-03-27 | 9618.8 | 256.7 | | 9800.0 | | 3400.0 | | 2/23/21 | |
| Bristol County | RI | 2020-03-25 | 2020-05-11 | 8458.6 | 252.8 | | 6146.1 | | 2873.6 | | 2/21/21 | |
| *Rural Counties with social and medical vulnerabilities* | | | | | |  | |  | |  | |  |
| Hancock County | GA | 2020-04-07 | 2020-04-28 | 10265.9 | 658.8 | | 15009.6 | | 4372.3 | |  | |
| Lamb County | TX | 2020-03-28 | 2020-07-16 | 14865.5 | 600.4 | | 13710.3 | | 7531.5 | | 2/23/21 | |
| McKinley County | NM | 2020-03-20 | 2020-04-04 | 16451.8 | 599 | | 18600.0 | | 7800.0 | | 2/23/21 | |
| Neshoba County | MS | 2020-03-27 | 2020-04-14 | 12793.1 | 566.5 | | 14303.9 | | - | | 2/23/21 | |
| Dawson County | TX | 2020-03-29 | 2020-04-09 | 13479.7 | 538.9 | | 7797.8 | | 2068.3 | | 2/23/21 | |
| East Feliciana Parish | LA | 2020-03-26 | 2020-04-08 | 15177.4 | 538.7 | | 11322.8 | | - | | 2/23/21 | |
| Glascock County | GA | 2020-05-07 | 2020-08-11 | 8146.9 | 534.2 | | 31652.8 | | 14991.7 | |  | |
| Big Horn County | MT | 2020-04-10 | 2020-06-05 | 17483.9 | 532.3 | | 19410.7 | | 8569.5 | | 2/23/21 | |
| Bienville Parish | LA | 2020-03-21 | 2020-03-25 | 11827.5 | 518.5 | | 12062.2 | | - | | 2/23/21 | |
| Wilcox County | GA | 2020-03-31 | 2020-04-18 | 8840.2 | 499.3 | | 7921.0 | | 4652.7 | |  | |
| *Older Rural counties with greater social assets* | | | | | |  | |  | |  | |  |
| Hamlin County | SD | 2020-03-28 | 2020-11-21 | 11242 | 621.8 | | 6856.5 | | 4843.7 | | 2/23/21 | |
| Grant County | SD | 2020-05-06 | 2020-09-12 | 13264.3 | 517.7 | | 6926.0 | | 6030.5 | | 2/23/21 | |
| Day County | SD | 2020-04-29 | 2020-10-17 | 11753 | 508.6 | | 10517.7 | | 8465.0 | | 2/23/21 | |
| Norton County | KS | 2020-05-01 | 2020-10-20 | 22357.3 | 497.2 | | 12044.2 | | - | |  | |
| Floyd County | TX | 2020-04-08 | 2020-06-12 | 12831.9 | 496.8 | | 14630.8 | | 5893.4 | | 2/23/21 | |
| Nemaha County | KS | 2020-04-28 | 2020-08-12 | 14377.2 | 492.4 | | 6312.2 | | 2067.9 | |  | |
| Rosebud County | MT | 2020-06-02 | 2020-06-26 | 12777.2 | 485.5 | | 21074.7 | | 7800.9 | | 2/23/21 | |
| Harrison County | IA | 2020-03-13 | 2020-08-09 | 11674 | 481.1 | | 9643.4 | | 3254.6 | |  | |
| Ida County | IA | 2020-05-12 | 2020-09-24 | 11723.4 | 467.8 | | 9823.1 | | 4180.7 | |  | |
| McCook County | SD | 2020-03-13 | 2020-04-09 | 13198.7 | 432.7 | | 8312.3 | | 7104.2 | | 2/23/21 | |
| *Urban Counties with social vulnerabilities* | | | | | |  | |  | |  | |  |
| Mifflin County | PA | 2020-03-29 | 2020-05-18 | 9737.8 | 359.1 | | 9363.5 | | 4262.0 | | 2/23/21 | |
| Northumberland County | PA | 2020-03-27 | 2020-05-18 | 8557 | 346.9 | | 10859.3 | | 5803.5 | | 2/23/21 | |
| Decatur County | IN | 2020-03-24 | 2020-03-31 | 10207.5 | 343.4 | | 14693.6 | | 10106.7 | |  | |
| Dakota County | NE | 2020-04-12 | 2020-04-25 | 19125.6 | 333.6 | | - | | - | |  | |
| Passaic County | NJ | 2020-03-08 | 2020-03-22 | 10933.2 | 327.4 | | 10520.6 | | 5176.7 | | 2/24/21 | |
| Essex County | NJ | 2020-03-12 | 2020-03-19 | 8821.7 | 327.3 | | 12198.3 | | 5352.1 | | 2/24/21 | |
| Monroe County | GA | 2020-03-20 | 2020-04-04 | 8317.6 | 316.1 | | 10352.5 | | 6319.0 | |  | |
| Knox County | IL | 2020-03-29 | 2020-07-22 | 9847.9 | 311.3 | | 24888.3 | | 7287.7 | |  | |
| Jefferson County | IL | 2020-04-06 | 2020-04-22 | 10312 | 304.1 | | 16208.4 | | 3651.5 | |  | |
| Habersham County | GA | 2020-03-27 | 2020-04-10 | 10934.6 | 301.8 | | 11987.8 | | 6166.8 | |  | |
